# DNA oxidation profiles of copper phenanthrene chemical nucleases

**DOI:** 10.3389/fchem.2015.00028

**Published:** 2015-04-21

**Authors:** Zara Molphy, Creina Slator, Chryssostomos Chatgilialoglu, Andrew Kellett

**Affiliations:** ^1^School of Chemical Sciences, National Institute for Cellular Biotechnology, Dublin City UniversityDublin, Ireland; ^2^Istituto per la Sintesi Organica e la Fotoreattività, Consiglio Nazionale delle RicercheBologna, Italy; ^3^Institute of Nanoscience and Nanotechnology, National Center for Scientific Research “Demokritos,”Athens, Greece

**Keywords:** copper, phenazine, chemical nuclease, hydroxyl radical, DNA damage, 8-oxo-dG

## Abstract

The deleterious effects of metal-catalyzed reactive oxygen species (ROS) in biological systems can be seen in a wide variety of pathological conditions including cancer, cardiovascular disease, aging, and neurodegenerative disorder. On the other hand however, targeted ROS production in the vicinity of nucleic acids—as demonstrated by metal-activated bleomycin—has paved the way for ROS-active chemotherapeutic drug development. Herein we report mechanistic investigations into the oxidative nuclease activity and redox properties of copper(II) developmental therapeutics [Cu(DPQ)(phen)]^2+^ (Cu-DPQ-Phen), [Cu(DPPZ)(phen)]^2+^ (Cu-DPPZ-Phen), and [{Cu(phen)_2_}_2_(μ-terph)](terph) (Cu-Terph), with results being compared directly to Sigman's reagent [Cu(phen)_2_]^2+^ throughout (phen = 1,10-phenanthroline; DPQ = dipyridoquinoxaline; DPPZ = dipyridophenazine; Terph = terephthalate). Oxidative DNA damage was identified at the minor groove through use of surface bound recognition elements of methyl green, netropsin, and [Co(NH_3_)_6_]Cl_3_ that functioned to control complex accessibility at selected regions. ROS-specific scavengers and stabilizers were employed to identify the cleavage process, the results of which infer hydrogen peroxide produced metal-hydroxo or free hydroxyl radicals (^•^OH) as the predominant species. The extent of DNA damage owing to these radicals was then quantified through 8-oxo-2′-deoxyguanosine (8-oxo-dG) lesion detection under ELISA protocol with the overall trend following Cu-DPQ-Phen > Cu-Terph > Cu-Phen > Cu-DPPZ. Finally, the effects of oxidative damage on DNA replication processes were investigated using the polymerase chain reaction (PCR) where amplification of 120 base pair DNA sequences of varying base content were inhibited—particularly along A-T rich chains—through oxidative damage of template strands.

## Introduction

Oxygen radical generation is an inevitable consequence of aerobic existence and has been implicated in a wide variety of pathological conditions including cancer, cardiovascular disease, aging, and neurodegenerative disease (Cooke and Evans, 2007). Reactive oxygen species (ROS) are created in a variety of endogenous chemical and biological processes in the human body—predominantly through oxygen metabolism. The sequential reduction of molecular oxygen can generate reactive intermediates such as superoxide (O^•−^_2_) and hydrogen peroxide (H_2_O_2_) that initiate a cascade of redox reactions toward the production of hydroxyl radicals (^•^OH) and related metal-oxo species (Kellett et al., 2012). Molecular targets of ROS include proteins, lipids, and nucleic acids—the deleterious effects of which include base and deoxyribose modifications that ultimately precipitate single or double strand breaks. To counteract this, the majority of cells possess defense mechanisms such as base excision repair (BER)—*e.g.*, 8-oxoguanine glycosylase (OGG1) (Xu et al., 2014)—and nucleotide excision repair (NER) pathways that prevents genome instability to ultimately limit cytotoxicity, the accumulation of deleterious mutations, and maintain genome integrity. Two major ^•^OH induced DNA lesions are 8-oxoguanine (8-oxo-dG), a mutagenic lesion that induces G → T transversions widely seen in mutated oncogenes and tumor suppressor genes, and the poorly mutagenic thymine glycol (Basu et al., 1989; Chatgilialoglu and O'Neill, 2001). Recent evidence suggests ^•^OH attacks occur primarily at base moieties and account for the majority of total hydrogen atom abstraction on DNA alone (Chatgilialoglu et al., 2011). Thus, 8-oxo-dG has been subjected to intensive investigation due to its prominence as a biomarker within ROS-mediated disease pathology, and its ease of detection in bodily fluids and tissue samples has allowed a variety of detection methods to accurately assess 8-oxo-dG lesions including high-pressure liquid chromatography (HPLC), gas chromatography (GC), mass spectrometry (MS), and the enzyme linked immunosorbent assay (ELISA).

In addition to the induction of endogenous DNA damage, exogenous sources including UV light, ionizing radiation, environmental agents, pharmaceuticals, and industrial chemicals can also initiate ROS production (Klaunig et al., 2010). Indeed the clinical antineoplastic agent bleomycin (BLM) is a redox active agent capable of DNA oxidative cleavage in the presence of Fe(II) (and Cu(I)), molecular oxygen, and endogenous one electron reductants (Stubbe and Kozarich, 1987; Burger, 1998; Chen et al., 2008). Bleomycin can abstract hydrogen atoms from deoxyribose in the DNA backbone, specifically from C4′ position (Breen and Murphy, 1995). The active form of Fe(II)-BLM is a ternary, high-valence Fe(III)-O^•^ species (Rodriguez and Hecht, 1982; Pratviel and Bernadou, 1989; Gajewski et al., 1991) that undergoes an electron reduction by biological reductants (*e.g.*, *L*-ascorbate) or by another molecule of Fe(II)-BLM (Burger et al., 1981; Natrajan et al., 1990). Fe(II)-BLM can form 8-oxo-dG and other base propenals, however these are known to occur in small amounts; the formation of such DNA degradation products results from ^•^OH oxidative damage—a side product only of the ferryl-oxo species—that does not functionally contribute to biological systems or participate in the nuclease activity of activated Fe(II)-BLM (Rodriguez and Hecht, 1982).

Our group have recently investigated a range of [Cu(phen)_2_]^2+^ (Cu-Phen) (Phen = 1,10-phenanthroline) type systems as potential lead compounds for therapeutic and biochemical application (Kellett et al., 2011; Prisecaru et al., 2012, 2013; Molphy et al., 2014). [Cu(phen)_2_]^2+^, originally reported by Sigman et al. (1979), is believed to cleave DNA through a variety of copper bound oxidants including Cu^3+^-OH and Cu^+^-OOH with the possibility of free ^•^OH playing a role in the overall process (Marshall et al., 1981; Johnson and Nazhat, 1987). Recent work on the development of bis-chelate Cu^2+^ phenanthroline-phenazine cationic complexes of [Cu(DPQ)(phen)]^2+^ (Cu-DPQ-Phen) and [Cu(DPPZ)(phen)]^2+^ (Cu-DPPZ-Phen) (DPQ = dipyridoquinoxaline; DPPZ = dipyridophenazine) have demonstrated how extension of the ligated phenazine ligand influences DNA recognition and oxidative degradation (Molphy et al., 2014). Indeed, when designer phenazine ligands (DPQ and DPPZ) are incorporated into the “copper bis-phen” chemical nuclease model, these agents display enhanced DNA recognition and intercalation among the highest reported on ctDNA (Table 1, *K*_app_ ≈ 3 × 10^7^ M(bp)^−1^). Since nuclearity is also established as an important factor in oxidative DNA cleavage (Li et al., 2005; van der Steen et al., 2010), we also reported the dinuclear complex, [{Cu(phen)_2_}_2_(μ-terph)](terph) (Cu-Terph) (terph = terephtalate), which is capable of inducing oxidative DNA strand breaks in the absence of exogenous reductant (Kellett et al., 2011). Cu-Terph has promising *in vitro* cytoxicity toward human derived breast, prostate, colon, ovarian, and lung human cance cell lines, with comparable activity to mitoxantrone—a clinincal anthracene topoisomerase II inhibitor (Kellett et al., 2011; Prisecaru et al., 2012).

**Table 1:** **Summary of DNA binding properties of tested complexes toward calf thymus DNA (ctDNA) along with synthetic nucleic acid polymers poly[d(A-T)_2_] and poly[d(G-C)_2_]**.

**Compound**	**C_50_^a^**	***K*_app_ M(bp)^−1^^b^**	***Q* (μM) poly[d(A-T)_2_]^c^**	***Q* (μM) poly[d(G-C)_2_]^c^**	**ΔT_*M*_ (°C) poly[d(A-T)_2_]^d^**	**ΔT_*M*_ (°C) poly[d(G-C)_2_]^d^**
Cu-Phen	179.21	0.67 × 10^6^	13.34	7.96	−0.02 ± 0.29	06.64 ± 1.58
Cu-DPQ-Phen	3.93	30.45 × 10^6^	8.34	3.97	0.60 ± 0.18	11.39 ± 1.10
Cu-DPPZ-Phen	4.63	25.85 × 10^6^	11.60	10.12	0.50 ± 0.10	10.44 ± 1.10
Cu-Terph	39.36	0.30 × 10^6^	8.6	10.3	NT	NT

a *C_*50*_ = concentration required to reduce 50% fluorescence of saturated bound ethidium bromide (12.6 μM) on ctDNA (10 μM)*.

b *K_app_ = K_e_ × 12.6/C_*50*_ where, K_e_ = 9.5 × 10^*6*^ M(bp)^−*1*^ (apparent binding constant on ctDNA)*.

c *Fluorescence Quenching (Q) of limited bound Ethidium Bromide (5 μM) bound poly[d(A-T)_*2*_] and poly[d(G-C)_*2*_] by Cu^*2*^+ complexes*.

d *ΔT_M_ = difference in thermal melting (T_M_) of drug-treated nucleotide at r = 0.1 compared with drug-untreated nucleotide*.

*NT = not tested*.

**Scheme 1:**
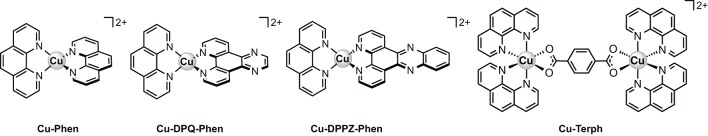
**Molecular structures of the copper(II) complex cations examined in this study**.

In this contribution we identify, using head-to-head analysis, the comparative oxidative DNA cleavage properties of DNA binding Cu^2+^ complexes Cu-Phen, Cu-DPQ-Phen, Cu-DPPZ-Phen, and Cu-Terph (Scheme 1) through a variety of biophysical and molecular biological methods. Additionally, we report these agents inhibit DNA polymerase activity—particularly at A-T rich sites—through oxidative degradation of template strands. To that end, we report (*i*.) oxidative DNA profiles in the presence of DNA recognition agents of netropsin, methyl green, and [Co(NH_3_)_6_]Cl_3_, (*ii*.) DNA cleavage profiles in the presence of radical trapping and stabilizing co-factors, (*iii*.) quantitation of 8-oxo-dG lesions arising from complex treated superhelical plasmid DNA, and (*iv*.) DNA polymerase inhibition on DNA templates of differential A-T content. The DNA binding profiles for this series have previous been reported and are summarized in Table 1 (Kellett et al., 2011; McCann et al., 2013; Prisecaru et al., 2013; Molphy et al., 2014); simple phenanthroline containing complexes (Cu-Phen and Cu-Terph) have moderate binding constants toward ctDNA while phenazine compounds (Cu-DPQ-Phen and Cu-DPPZ-Phen) can be considered as high-affinity dsDNA intercalators. Further, ethidium bromide fluorescence quenching on alternating duplex polymers—poly[d(A-T)_2_] and poly[d(G-C)_2_]—has shown complexes intercalate from both minor and major grooves. It has not been established, as yet, if chemical nuclease activity occurs preferentially at either or both recognition sites.

## Materials and methods

### Preparation of the complexes

Chemicals were purchased from Sigma-Aldrich Ireland and used without further purification.

DPQ and DPPZ ligands were initially generated through the Schiff base condensation reactions of 1,10-phenanthroline-5,6-dione with ethylenediamine and *o*-phenylenediamine respectively. The bis-phenanthroline complex [Cu(phen)_2_](NO_3_)_2_ (Cu-Phen) was prepared by refluxing 1,10-phenanthroline with copper(II) nitrate in a 2:1 molar ratio in aqueous-ethanol (Prisecaru et al., 2013). The phenazine complexes [Cu(DPQ)(Phen)](NO_3_)_2_ (Cu-DPQ-Phen) and [Cu(DPPZ)(Phen)](NO_3_)_2_ (Cu-DPPZ-Phen) were prepared by treating the mono-phenanthroline complex [Cu(Phen)](NO_3_)_2_ with 1 molar equivalent of the corresponding phenazine ligand in ethanol (Molphy et al., 2014). The [Cu_2_(μ-terephthalate)(1,10-phen)_4_]^2+^ was prepared by ethanolic reflux of copper(II) terephthalate hydrate and 1,10-phenanthroline in a 1:2 ratio according to the reported method (Kellett et al., 2011).

### DNA cleavage studies

#### DNA cleavage in the presence of added reductant

The ability of the complexes to oxidatively damage DNA in the presence of added reductant was determined using a method previously published by this laboratory with minor changes (Molphy et al., 2014). Reactions were carried out according to the following general procedure: in a total volume of 20 μL using 80 mM HEPES buffer (pH 7.2) with 25 mM NaCl, 1 mM Na-L-ascorbate, 400 ng superhelical pUC19 (NEB, N3041) and varying concentrations of test complex (250 nM, 500 nM, 1 μM and 2.5 μM). Complexes were initially prepared in DMF and further diluted in HEPES buffer (Fisher). Samples were incubated at 37°C for 30 min. Reactions were quenched by adding 6× loading buffer (Fermentas) containing 10 mM Tris-HCl, 0.03% bromophenol blue, 0.03% xylene cyanole FF, 60% glycerol, 60 mM EDTA and samples were loaded onto an agarose gel (1.2%) containing 8 μL EtBr. Electrophoresis was completed at 70 V for 2 h in 1× TAE buffer.

#### DNA cleavage in the presence of non-covalently bound recognition elements

This protocol was adapted from a previously reported procedure (Tabassum et al., 2012). Briefly, 400 ng pUC19 was incubated with 25 mM NaCl, 1 mM Na-L-ascorbate, and 8, or 16 μM of either methyl green, netropsin or hexamine cobalt(III) chloride in 80 mM HEPES buffer (pH 7.2) for 45 min at 37°C. Sample tubes were then vortexed and varying concentrations of test complex were added (250 nM, 500 nM, 1 μM, and 2.5 μM). The reaction mixture was further incubated at 37°C for 30 min. The reaction was then quenched with 6× loading buffer and subjected to gel electrophoresis (prepared and stained as previously described).

#### DNA oxidation with ROS scavengers and stabilizers

The presence of ROS specific scavengers was used to determine the effect on the DNA cleavage abilities of each copper complex. The procedure was adapted to the previously reported method (Zhou et al., 2014). Briefly, to a final volume of 20 μL, 80 mM HEPES (pH = 7.2), 25 mM NaCl, 1 mM Na-L-ascorbate, and 400 ng of pUC19 DNA were treated with drug concentrations of 250 nM, 500 nM, 1 μM, and 2.5 μM in the presence ROS scavengers / stablilisers; KI (10 mM), NaN_3_(10 mM), DMSO (10%), and D_2_O (77%). Reactions were incubated for 30 min at 37°C, quenched with DNA loading dye and loaded onto 1.2% agarose gel and run under conditions previously described.

### HT quantitation of 8-oxo-dG

Quantitation of 8-oxo-dG lesions present in 3000 ng pUC19 plasmid DNA pre-incubated with test complexes (10 and 20 μM) at 37°C for 30 min was achieved utilizing a high throughput 8-oxo-dG ELISA kit (Trevigen) and performed as per manufacturers guidelines. Samples of damaged DNA were examined in triplicate using a 96 well plate, pre-coated with 8-oxo-dG along with varying concentrations of a standard 8-oxo-dG (200, 100, 50, 25, 12.5, 6.25, and 3.13 nM). An 8-oxo-dG monoclonal antibody, which competitively binds to 8-oxo-dG immobilized to each well, was added to the plate with excess antibody being washed with PBST (1× PBS, 0.1% Tween 20). The concentration of 8-oxo-dG was determined based on antibody retention in each well using goat anti-mouse IgG-HRP conjugated antibody and colorimetric detection substrate TACS-Sapphire. Product formation was inversely proportional to 8-oxo-dG present in the DNA sample. Samples were determined using a Bio-Tek synergy HT multimode microplate reader at 450 nm and quantitation of 8-oxo-dG was extrapolated from the standard curve.

### PCR inhibition studies

This protocol was adapted from a previously reported procedure (Sanchez-Cano et al., 2010). 400 ng pUC19 DNA was initially exposed to each test complex in the presence and absence of 1 mM added reductant at 37°C for 30 min (Figures S-2, S-3). 20 ng of damaged DNA template was removed and PCR reactions (35 cycles) were carried out with each varying G-C content primer set (Figure S-4) at optimum annealing temperatures and analyzed using gel electrophoresis. This investigation was replicated in the absence of added reductant (Figure S-5) and also at lower drug loading (250 nM, 500 nM, 1 μM, and 2.5 μM) with 1 mM reductant (Figure S-6).

## Results and discussion

### DNA cleavage in the presence of non-covalently bound recognition elements

**Figure 1:**
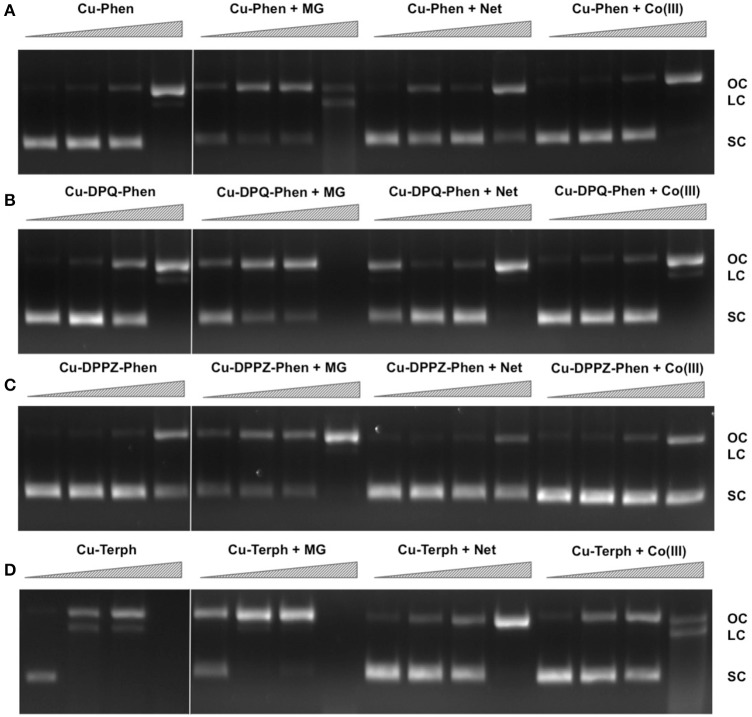
**Lane 1–4 (A–D) DNA cleavage reactions with 250 nM, 500 nM, 1.0 μM, and 2.5 μM test complex (A) Cu-Phen, (B) Cu-DPQ-Phen, (C) Cu-DPPZ-Phen, and (D) Cu-Terph, 400 ng superhelical pUC19 and 1 mM added Na-L-ascorbate incubated at 37°C for 30 min**. Lanes 5–16 **(A–D)** DNA cleavage reactions in the presence of recognition elements, methyl green (MG), netropsin (Net), and [Co(NH_3_)_6_]Cl_3_ (Co(III)), where 400 ng pUC19 was initially pre-treated with 8 μM of respective non-covalent binding control at 37° C for 45 min and then with 250 nM, 500 nM, 1 μM, and 2.5 μM test complex in the presence of 1 mM added Na-L-ascorbate at 37°C for 30 min.

We have previously shown that DNA oxidative cleavage by copper complexes is dependent on a range of factors including (but not limited to): plasmid DNA conformation and type, presence of competing metal chelating agents (*e.g.*, EDTA), reaction/exposure time, and presence/concentration of exogenous reductant or oxidant. In the current work we examine chemical nuclease activity of supercoiled pUC19 plasmid DNA in the presence of 1 mM reductant (Na-L-ascorbate) using agarose gel electrophoresis. In order to ensure the copper(I) active species (i.e., the nuclease) was fully generated, each complex was initially reduced with 1 mM of added reductant prior to pUC19 titration. Relaxation of supercoiled (SC, FI) pUC19 DNA into open circular (OC, FII) and linear (LC, FIII) conformations was employed to qualitatively measure the cleavage efficiency of complexes over a concentration range of 250 nM, 500 nM, 1.0 μM, and 2.5 μM for 30 min at 37°C (Figures 1A–D, lanes 1–4). Complexes show concentration-dependent relaxation of FI (superhelical) to FII (open circular/nicked), while FIII (linear conformation) is evident at 2.5 μM Cu-DPQ-Phen exposure and with 500 nM of the dinuclear agent Cu-Terph. Complete digestion of SC DNA occurs only with the maximum tested concentration (2.5 μM) of Cu-Terph. The overall trend in chemical nuclease activity is Cu-Terph > Cu-DPQ-Phen > Cu-Phen > Cu-DPPZ-Phen. The activity profiles observed here are in good agreement with those previously reported by this group; Cu-Terph has previously displayed nicking at 1.0 μM on the plasmid pBR322 with complete digestion occurring thereafter (Prisecaru et al., 2012). We also established that both 2.5 and 5.0 μM of Cu-Phen induced nicking (OC) on both pBR322 (Prisecaru et al., 2012; McCann et al., 2013) with activity being impeded in the presence of EDTA (Prisecaru et al., 2013). The nuclease activity of both Cu-DPQ-Phen and Cu-DPPZ-Phen has been identified previously (Molphy et al., 2014), however, direct analysis with the current conditions cannot be made.

In an attempt to determine DNA cleavage site specificity, minor groove (netropsin, Net) and major groove (methyl green, MG) binders, along a surface electrostatic binding and condensing agent ([Co(NH_3_)_6_]Cl_3_, Co(III)) were pre-incubated with pUC19 DNA prior to the addition of test complex (Figures 1A–D, lanes 5–16). In all cases, presence of the major groove binder MG enhanced chemical nuclease activity with greater nicking (OC) and linearization (LC) frequency compared with control experiments. Conversely, the minor groove binder Net impedes chemical nuclease activity as pUC19 is clearly protected from both OC and LC damage across all experiments. The cationic surface binding agent [Co(NH_3_)_6_]^3+^ had no major impact on the chemical nuclease activity of Cu-Phen and Cu-DPPZ-Phen but did reduce nicking by Cu-DPQ-Phen at 1 μM and was also effective in protecting pUC19 damage by Cu-Terph. Taken together, evidence here points toward the minor groove as the major site of DNA oxidation by this complex series; MG bound pUC19 primes the minor groove for chemical nuclease activity while titrated Net clearly diminishes this damage. Indeed this observation of minor groove targeting is consistent with previous analysis on the rapid cleavage of poly(dA-dT) by 2:1 phenanthroline-Cu^+^ mixtures (Sigman et al., 1979).

### DNA oxidation with ROS scavengers and stabilizers

**Figure 2:**
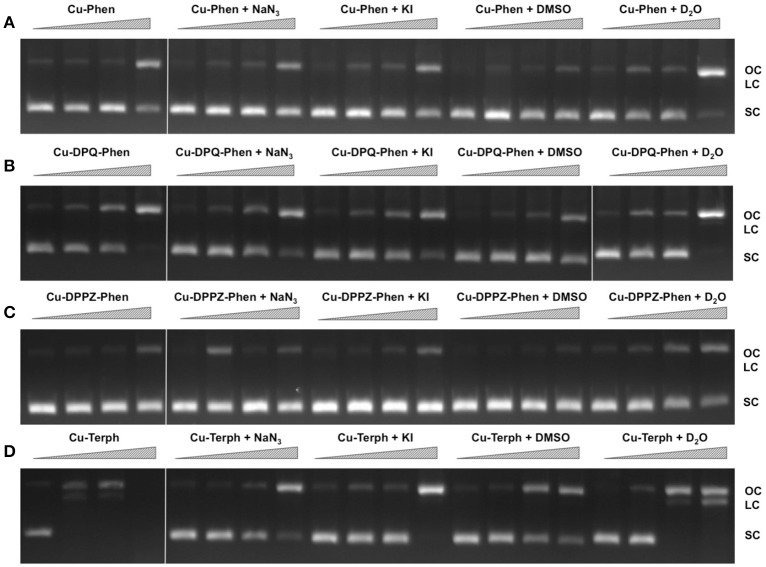
**DNA cleavage reactions in the presence of ROS scavengers**. 400 ng of SC pUC19 was incubated for for 30 min at 37°C with 250 nM, 500 nM, 1 μM, and 2.5 μM of test complex **(A)** Cu-Phen, **(B)** Cu-DPQ-Phen, **(C)** Cu-DPPZ-Phen, and **(D)** Cu-Terph in the presence of 1 mM added Na-L-ascorbate for 30 min. Lanes 1–4 metal complex only, lanes 5–8, complex + 10 mM NaN_3_; lanes 9–12, complex + 10 mM KI; lanes 13–16, complex + 10% DMSO; and lanes 17–20, complex + 77% D_2_O.

In order to examine ROS species involved in DNA oxidation, activity was investigated in the presence of radical-specific scavengers and stabilizers (Table 2) with results shown in Figure 2. Before complex analysis, scavengers were confirmed to have no impact on pUC19 conformation (data not shown). Control experiments are in excellent agreement with those observed in Figure 1 (lanes 1–4), however, a fraction of superhelical (FI) pUC19 was found to remain upon 2.5 μM exposure of Cu-Phen. Results here suggest that ^•^OH is the most prevalent radical species involved in strand scission as the presence of DMSO considerably impedes cleavage activity of all complexes. It is noteworthy DMSO had a major impact on cleavage activity of Cu-Terph as only the maximum tested concentrations (1.0 and 2.5 μM) contained nicked cleavage products. The presence of the H_2_O_2_ scavenger, KI, was also found to inhibit chemical nuclease activity of tested complexes—again most notably within Cu-Terph reactions—and this observation is consistent with previous trapping studies conducted on these model systems (Johnson and Nazhat, 1987; Prisecaru et al., 2013). It is interesting to note the catalase enzyme is a more effective scavenger of H_2_O_2_ compared with KI as previous work revealed complete inhibition of DNA oxidation by Cu-Phen, Cu-DPQ-Phen, and Cu-DPPZ-Phen complexes (Molphy et al., 2014).

**Table 2:** **Scavengers and stabilizers utilized within this study**.

**Scavenger^a^/Stabilizer^b^**	**ROS**	**References**
NaN_3_^a^	^1^O_2_	Franco et al., 2007
KI^a^	H_2_O_2_	Dunand et al., 2007; Steffens et al., 2012
DMSO^a^	^•^OH	Franco et al., 2007; Mazzer et al., 2007
D_2_O^b^	^1^O_2_	Merkel et al., 1972; Xia et al., 2006

The role of ^1^O_2_ was next examined utilizing the NaN_3_ scavenger (Franco et al., 2007) and D_2_O as a ^1^O_2_ stabilizer (Merkel et al., 1972; Xia et al., 2006). Nuclease activity by Cu-Phen, Cu-DPQ-Phen, and Cu-DPPZ-Phen complexes was only marginally inhibited by NaN_3_ while no change in activity (relative to control) was observed in D_2_O thus suggesting a limited role in DNA oxidation by ^1^O_2_. NaN_3_ did, however, impede activity by Cu-Terph but the role of ^1^O_2_ in the scission process could not be verified as D_2_O was also found to reduce activity, somewhat.

### Quantitation of 8-oxo-dG

**Figure 3:**
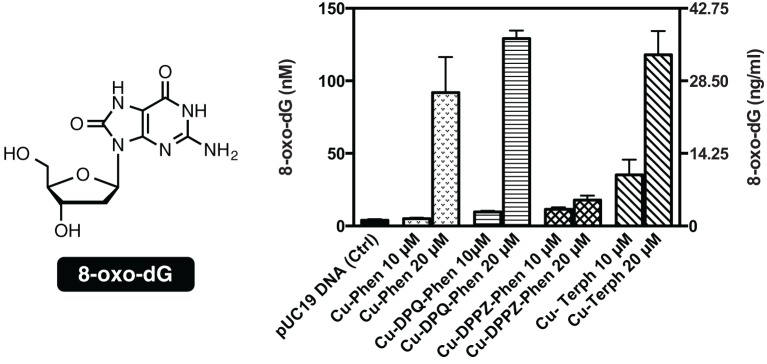
**Structure and quantification of 8-oxo-dG**. Graph represents level of generated 8-oxo-dG as nM (left axis) and ng/mL (right axis). 3000 ng of SC pUC19 with 10 and 20 μM of test complexes Cu-Phen, Cu-DPQ-Phen, Cu-DPPZ-Phen, and Cu-Terph with 1 mM Na-L-ascorbate were incubated at 37°C for 30 min and followed by ELISA protocol.

To examine if the oxidative DNA lesion, 8-oxo-dG, is formed during complex reactions with DNA, an HT 8-oxo-dG ELISA kit was employed for immunological detection and quantification. The complex series was investigated at 10 and 20 μM with 3000 ng of SC pUC19, with 8-oxo-dG being quantified in both nM and ng/mL (Figure 3). Nuclease activity was firstly confirmed at 10 μM and 20 μM (Figure S-1). Low numbers of lesions were detected in the untreated control (3.92 ± 0.79 nM) and the exposure of pUC19 to each of the complexes at 10 μM resulted in detectable increases in 8-oxo-dG (between 35.24 and 5.05 nM) with the trend following Cu-Terph > Cu-DPPZ-Phen > Cu-DPQ-Phen > Cu-Phen. Upon 20 μM complex exposure significant levels of 8-oxo-dG (between 129.22 and 17.77 nM) were produced with the trend changing toward Cu-DPQ-Phen > Cu-Terph > Cu-Phen > Cu-DPPZ-Phen. Given the ^•^OH radical is fundamental in the production of 8-oxo-dG, results here demonstrate DNA oxidation by copper phenanthrene complexes, particularly under extensive shearing conditions (Figure S-1), drive formation of 8-oxo-dG lesions.

### PCR inhibition studies

**Figure 4:**
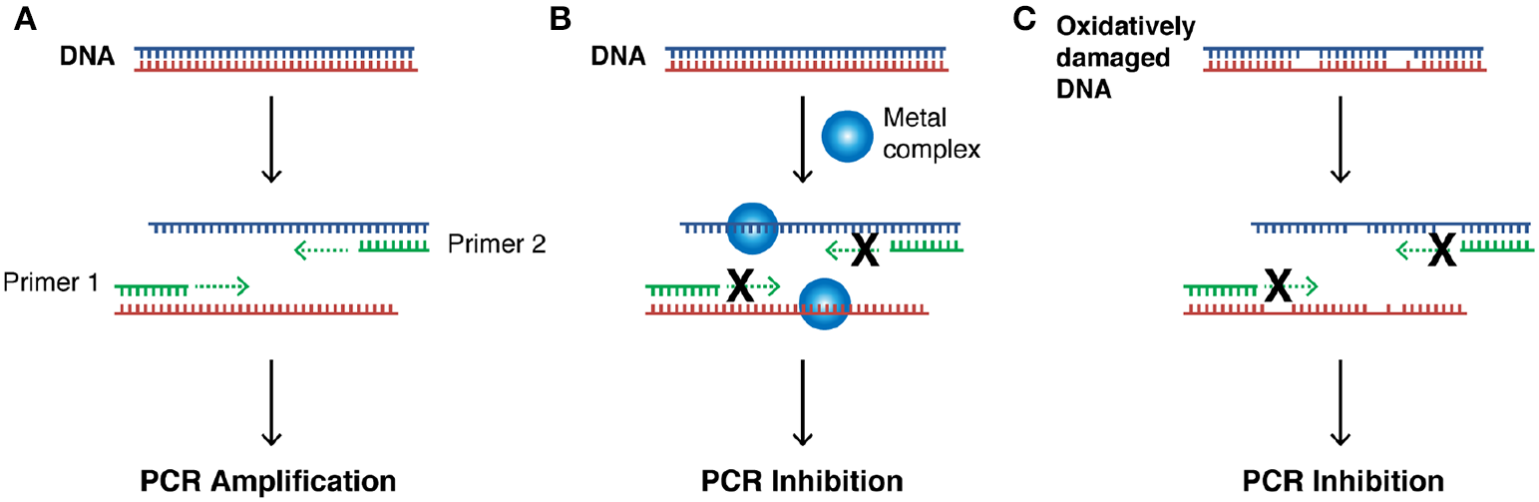
**(A)** Illustration of steps involved in a successful PCR reaction (denaturation, primer annealing, primer extension, and template amplification), **(B)** the impact of a bound metal complex as physical block of the primer extension step, **(C)** inhibition of DNA amplification in the PCR cycle through the oxidative damage of template strand.

**Figure 5:**
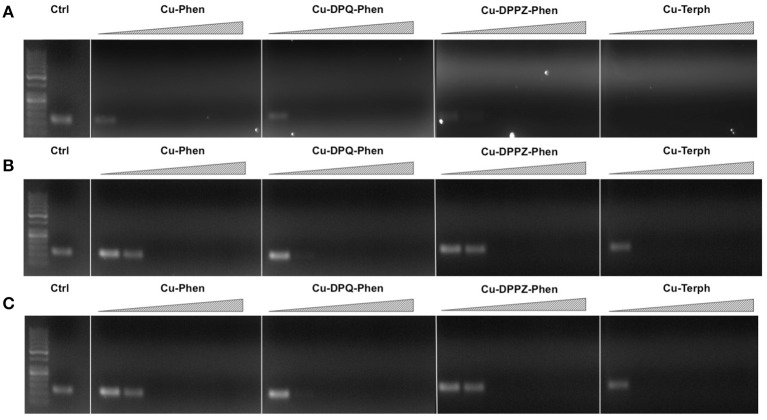
**400 ng pUC19 DNA was initially exposed to 2.5, 5, 10, 20, 30, 40, and 50 μM of each test complex in the presence of 1 mM added reductant at 37°C for 30 min**. 20 ng of damaged DNA template was removed and PCR reaction was carried out with each varying GC content primer set at optimum annealing temperatures and analyzed using gel electrophoresis. Panel **(A)** Lane 1, 50 bp DNA ladder, lane 2, 35 % GC control; lane 3–9, 35% GC + Cu-Phen; lane 10–16, 35% GC + Cu-DPQ-Phen; lane 17–23, 35% GC + Cu-DPPZ-Phen and lane 24–30, 35% GC + Cu-Terph. Panel **(B)** 50% GC, and Panel (C) 63% GC respectively. All duplex sequences generated were 120 base pairs.

Our next aim was to investigate how oxidative lesions—induced through complex exposure—can ultimately impact on *in vitro* DNA processing by the polymerase chain reaction (PCR) (Figure 4). During the normal PCR process, a DNA template is initially denatured through heating to more than 90°C to separate double stranded DNA into constituent single strands. The second step involves lowering the temperature (40–60°C) to allow specifically designed forward and reverse primers to anneal at targeted regions (for selective amplification) through complementary base pairing. At this point the temperature is increased again to allow Taq polymerase to attach at each priming site and extend to synthesize a new DNA strand. This thermal cycling process allows for a chain reaction to occur in which the selected DNA template is exponentially amplified creating millions of copies of the targeted sequence (Figure 4A). In our study 400 ng of pUC19 plasmid DNA was initially exposed to increasing concentrations (2.5, 5, 10, 20, 30, 40, and 50 μM) of test complexes in the absence and presence of exogenous reductant at 37°C for 30 min and used as a substrate for the PCR reaction along with specific primer sets to generate three 120 bp sequences of varying G·C content (35, 50, and 63%). PCR inhibition (up to 50 μM) was not achieved by any tested agent (Figure S-5) in the absence of added reductant indicating physical blocking of the PCR process (Figure 4B) does not occur. With added reductant, however, the pattern emerges as described in Figure 4C. In the high A·T amplification set (35% G·C), PCR was inhibited by 5.0 μM of the mono-nuclear complexes (Cu-Phen, Cu-DPQ-Phen and Cu-DPPZ-Phen). In the case of the di-nuclear agent (Cu-Terph), complete inhibition of template amplification was observed at all exposure levels (Figure 5A). Within the 50 and 63% G·C templates (Figures 5B,C respectively), the PCR reaction was impeded at 5.0 μM for both Cu-DPQ-Phen and Cu-Terph complexes whereas template DNA, oxidatively damaged by 5.0 μM of Cu-Phen and Cu-DPPZ-Phen, was still suitable for amplifying 120 base pair DNA sequences.

## Conclusions

Mechanistic investigations into oxidative cleavage properties of the copper(II) complex series [Cu(phen)_2_]^2+^ (Cu-Phen) [Cu(DPQ)(phen)]^2+^ (Cu-DPQ-Phen), [Cu(DPPZ)(phen)]^2+^ (Cu-DPPZ-Phen), and [{Cu(phen)_2_}_2_(μ-terph)](terph) (Cu-Terph) reveal chemical nuclease activity occurs primarily at the minor groove; titration of the major groove binder, methyl green, enhances DNA degradation—most likely by directing (priming) complex-DNA interactions to the minor groove—while the presence of the minor grove binder, netropsin, was found to significantly reduce oxidative damage on pUC19. It is also interesting to note that no correlation exists between chemical nuclease activity (Figure 1) and apparent DNA binding constant (Table 1). Instead, nuclearity has a more dramatic effect as evidenced by Cu-Terph mediated DNA damage. ROS-specific scavengers employed to identify the cleavage mechanism revealed metal-hydroxo or free hydroxyl radicals (^•^OH), and not ^1^O_2_, as the predominant species generated; DMSO was found to limit DNA oxidation—most likely through the trapping of hydroxyl radicals [(CH_3_)_2_SO + ^•^OH → CH_3_SO_2_H + CH_3_] (Burkitt and Mason, 1991) —with sodium azide (NaN_3_) having neglibile influence on all complexes except Cu-Terph. It is also likely that hydrogen peroxide (H_2_O_2_) is the key intermediary in ^•^OH production as the peroxide scavenger KI (Dunand et al., 2007; Steffens et al., 2012) was refractory to oxidative damage by tested agents. The generation of hydroxyl-based radicals was corroborated through identification of 8-oxo-2′-deoxyguanosine (8-oxo-dG) DNA lesions quantified under an ELISA protocol. 8-oxo-dG liberation followed the overall trend Cu-DPQ-Phen > Cu-Terph > Cu-Phen > Cu-DPPZ with higher lesion numbers detected under heavily sheared (damaged) plasmid conditions. Finally, oxidative damage by the complex series was found to inhibit the DNA replication process; polymerase chain reaction (PRC) reactions were impeded—particularly along A-T rich chains—through oxidative damage of template strands with the di-nuclear Cu-Terph, and mono-nuclear Cu-DPQ-Phen, being particularly potent oxidants to this process.

### Conflict of interest statement

The authors declare that the research was conducted in the absence of any commercial or financial relationships that could be construed as a potential conflict of interest.
