# Supplementary material for: DNA oxidation profiles of copper phenanthrene chemical nucleases
Source: Front Chem. 2015 Apr 21;3:28. doi: 10.3389/fchem.2015.00028 (PMC4404973; doi:10.3389/fchem.2015.00028)
Supplement: Supplementary file 1 [file DataSheet1.DOCX]

**Electronic Supporting Information**

Accompanying the manuscript

**DNA Oxidation Profiles of Copper Phenanthrene Chemical Nucleases**

Zara Molphy,^1^ Creina Slator,^1^ Chryssostomos Chatgilialoglu,^2,3^ and Andrew Kellett^1*^

**List of Content:**

**S-1: Corresponding nuclease activity for 8-oxo-dG conditions**

**S-2: DNA cleavage optimisation (high concentration range with added reductant) for PCR amplification studies**

**S-3: DNA cleavage optimisation (high concentration range without added reductant) for PCR amplification studies**

**S-4: PCR primer design**

**S-5: PCR amplification studies without reductant (high concentration range)**

**S-6: PCR amplification studies with reductant (low concentration range)**

**S-1: Corresponding nuclease activity for 8-oxo-dG conditions**


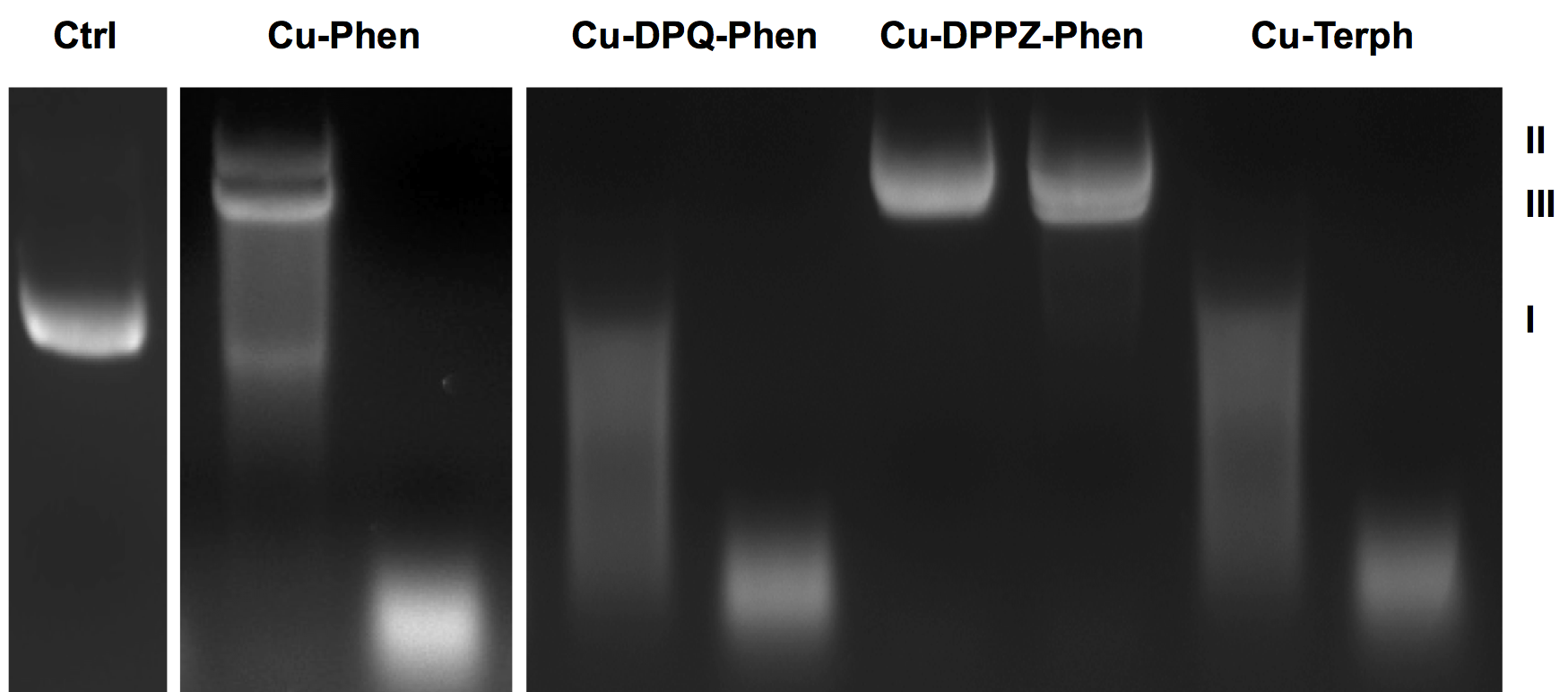


**Figure S-1.** DNA cleavage reactions with 10 and 20 μM test complex, 3000 ng superhelical pUC19 and 1 mM added Na-L-Ascorbate incubated at 37 °C for 30 minutes. Lane 1: pUC19 only, lane 2,3: 10 and 20 μM Bis-Phen, lane 4,5: 10 and 20 μM Cu-DPQ-Phen, lane 6,7: 10 and 20 μM Cu-DPPZ-Phen, lane 8-9: 10 and 20 μM Cu-Terph.

**S-2: DNA cleavage optimisation (high concentration range with added reductant) for PCR amplification studies**


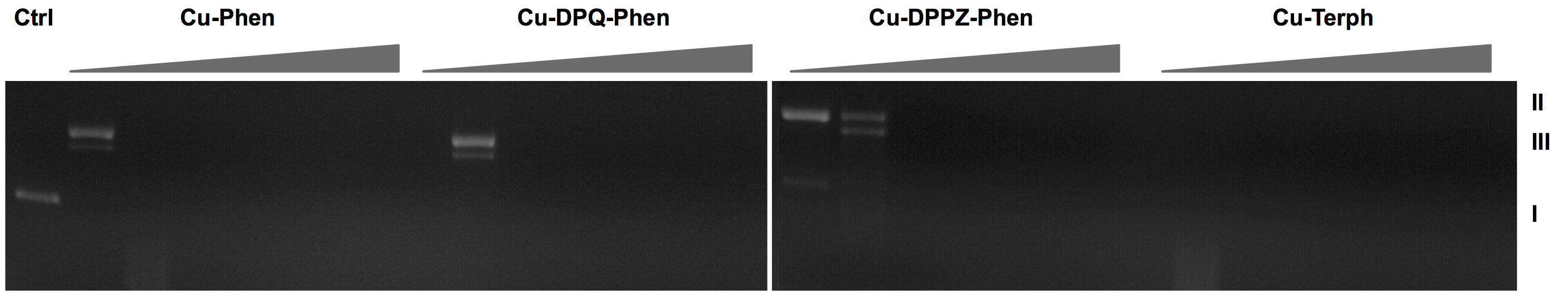


**Figure S-2.** DNA cleavage reactions with 2.5, 5, 10, 20, 30, 40 and 50 µM test complex, 400 ng superhelical pUC19 and 1 mM added Na-L-Ascorbate incubated at 37 °C for 30 minutes. Lane 1: pUC19 only, lane 2-8: Bis-Phen and lane 9-15: Cu-DPQ-Phen, lane 16-22: Cu-DPPZ-Phen and lane 23-29: Cu-Terph.

**S-3: DNA cleavage optimisation (high concentration range without added reductant) for PCR amplification studies**


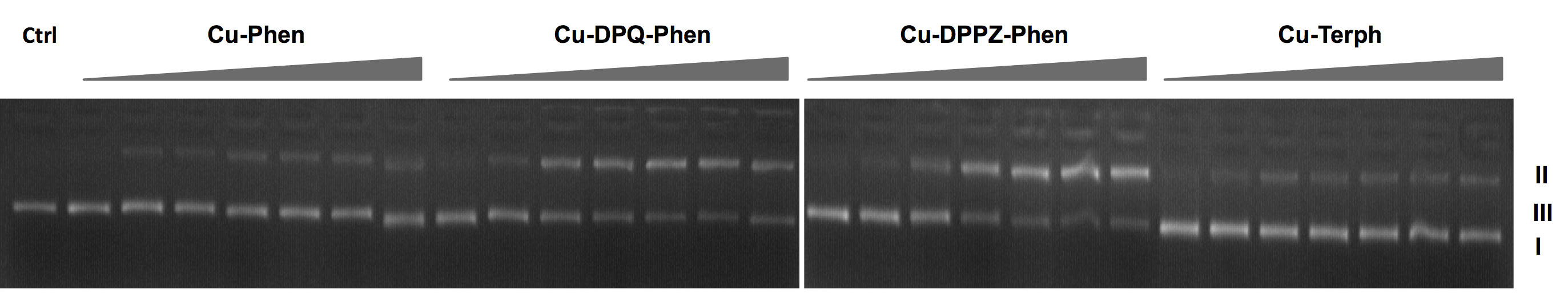


**Figure S-3.** DNA cleavage reactions with 2.5, 5, 10, 20, 30, 40 and 50 µM test complex, 400 ng superhelical pUC19 without added Na-L-Ascorbate incubated at 37 °C for 30 minutes. Lane 1: pUC19 only, lane 2-8: Bis-Phen and lane 9-15: Cu-DPQ-Phen, lane 16-22: Cu-DPPZ-Phen and lane 23-29: Cu-Terph.

**S-4: PCR Primer Design**

The pUC19 vector (2686 bp) was studied in detail and 3 sets of primers were designed such that it was possible to generate 120 bp long sequences of varying G·C content (35%, 50% and 63%). The lengths of the short nucleotide sequences were verified by carrying out PCR reactions (35 cycles) with 1 ng pUC19 plasmid using 2× MyTaq Red Mix (Bioline) at suitable annealing temperatures for respective primer pairs and comparing the band generated by gel electrophoresis to a 50 bp DNA ladder (Fermentas).

35% forward: 5’-gatcttttctacggggtctg-3’

35% reverse: 5’-gatttaaaacttcattttta-3’

50% forward: 5’-ttatcgccactggcagcagc-3’

50% reverse: 5’-accaaatactgttcttctag-3’

63% forward: 5’-tcgcgcgtttcggtgatgacg-3’

63% reverse: 5’-cacccgctgacgcgccctgacg-3’

**S-5: PCR amplification studies without reductant (high concentration range)**


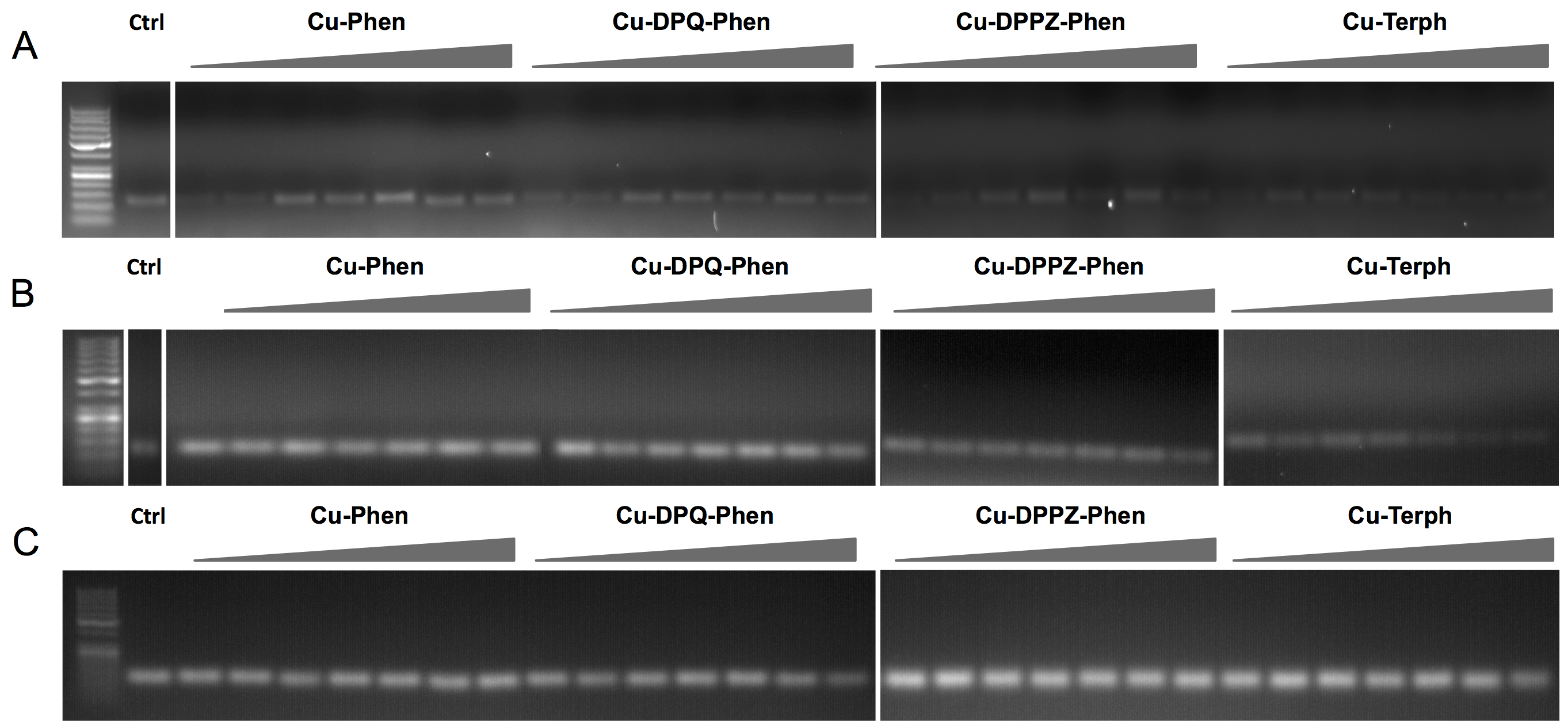


**Figure S-5.** 400 ng pUC19 DNA was initially exposed to 2.5, 5, 10, 20, 30, 40 and 50 µM of each test complex in the absence of added reductant at 37 °C for 30 minutes. 20 ng of complex exposed DNA template was removed from the reaction and PCR reaction was carried out as previously described with each varying G·C content primer set at optimum annealing temperatures and analysed using gel electrophoresis. Fig. A Lane 1: 35% G·C control, lane 2-8 35% G·C + Cu-Phen, lane 9-15: 35% G·C + Cu-DPQ-Phen and lane 16-22: 35% G·C + Cu-Terph. (B) 50% G·C and (C) 63% G·C respectively. All sequences generated were 120 base pairs.

**S-6: PCR amplification studies with reductant (low concentration range)**


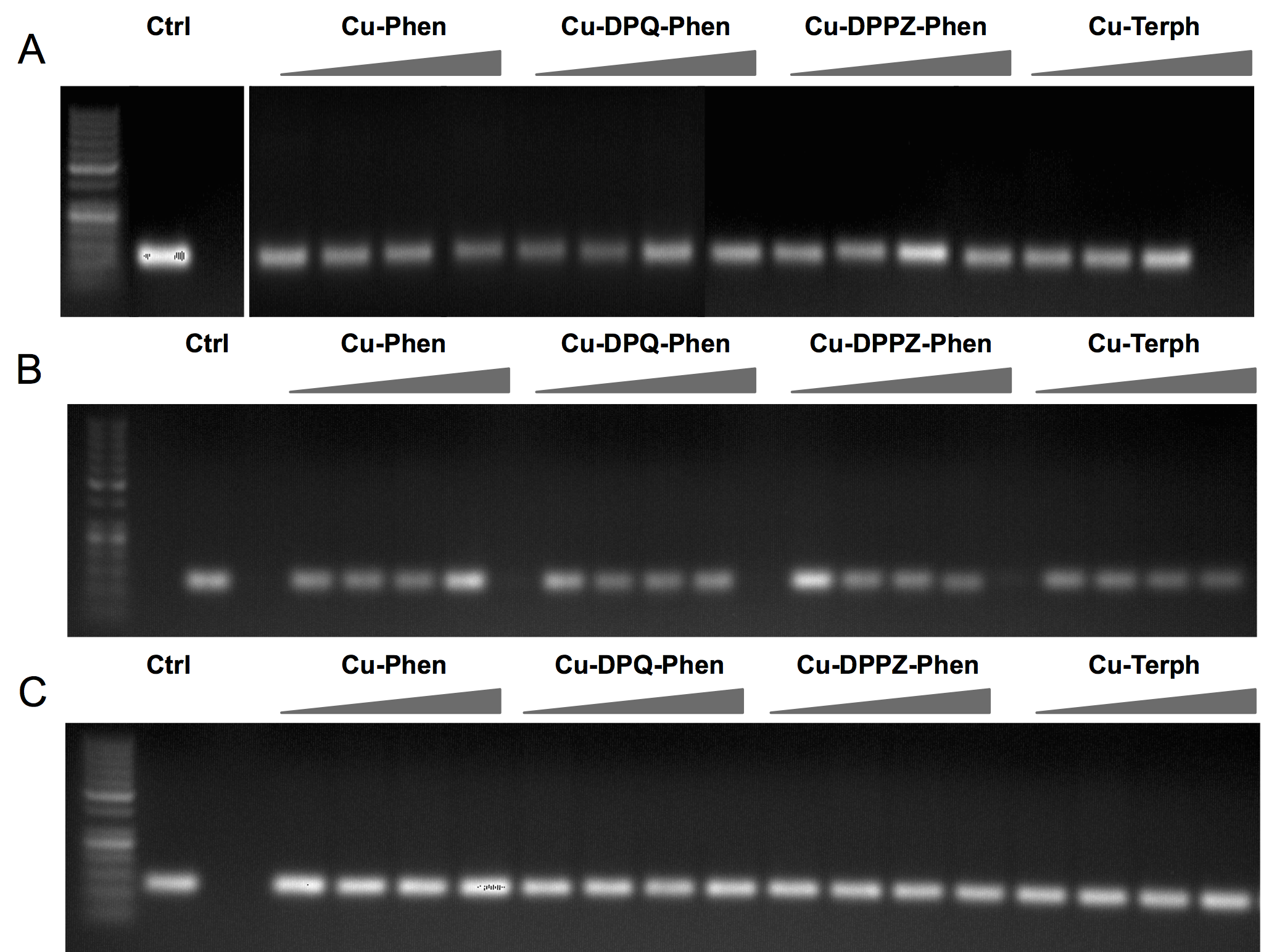


**Figure S-6.** 400 ng pUC19 DNA was initially exposed to 250 nM, 500 nM, 1 µM and 2.5 µM of each test complex in the presence of 1 mM added reductant at 37 °C for 30 minutes. 20 ng of complex exposed DNA template was removed from the reaction and PCR reaction was carried out as previously described with each varying G·C content primer set at optimum annealing temperatures and analysed using gel electrophoresis. Fig. A Lane 1: 35% G·C control, lane 2-5 35% G·C + Cu-Phen, lane 6-9 35% G·C + Cu-DPQ-Phen and lane 10-13: 35% G·C + Cu-Terph. (B) 50% G·C and (C) 63% G·C respectively. All sequences generated were 120 base pairs.
